# Eruptive Spitz nevus, a striking example of benign metastasis

**DOI:** 10.1038/s41598-020-73264-0

**Published:** 2020-10-01

**Authors:** Shyam S. Raghavan, Elisa S. Kapler, Martin M. Dinges, Boris C. Bastian, Iwei Yeh

**Affiliations:** 1 Department of Pathology, University of Virginia, Charlottesville, VA USA; 2 Colorado Permanente Medical Group, Denver, CO USA; 3 Department of Dermatology, University of California San Francisco, 1701 Divisadero St. Ste. 280, San Francisco, CA 94143 USA; 4 Department of Pathology, University of California San Francisco, 1701 Divisadero St. Ste. 280, San Francisco, CA 94143 USA

**Keywords:** Metastasis, Cancer, Melanoma

## Abstract

Metastasis is generally considered a characteristic of malignant tumors. Herein, we describe a patient with more than one hundred discrete Spitz nevi scattered all over her skin. Molecular analysis from three of the lesions identified a ROS1 fusion oncogene with identical genomic breakpoints, indicating that the nevi arose from a single transformed melanocyte and then disseminated throughout the integument. The demonstration of widespread distribution of a benign tumor with limited proliferative capability indicates that metastatic dissemination is not contingent on full malignant transformation. Thus, eruptive Spitz nevus is a striking example of benign metastasis, demonstrating that metastasis can occur before malignant transformation.

## Introduction

Spitz nevi are benign melanocytic neoplasms with distinct histopathological features, named after the pathologist Sophie Spitz^1^. They harbor a diverse set of initiating oncogenic alterations distinct from other types of nevi, including mutations in *HRAS*, oncogenic fusions involving the serine/threonine kinases *BRAF* or *MAP3K8* or receptor tyrosine kinases *ALK*, *ROS1*, *NTRK1*, *NTRK3*, *MERTK*, *MET*, or *RET*^2–7^. Spitz nevi are more common in children but can arise throughout life. They typically present as skin-colored, pink, brown or black dome shaped lesions that may regress over time^8^. While they most commonly occur as solitary lesions, they can occasionally present in an agminated (grouped) or disseminated fashion. The very rare setting in which disseminated Spitz nevi arise suddenly has been termed eruptive Spitz nevi and mostly affects young to middle age adults^9,10^.

The etiology of eruptive Spitz nevi is unknown. One proposed mechanism is that eruptive Spitz nevi result from immunosuppression and they have been reported to occur in this context^11,12^. This mechanism presupposes that the immune system actively prevents the formation of clinically detectable nevi after melanocytes acquire an initiating mutation. In this setting, immunosuppression would allow the rapid evolution of multiple Spitz nevi originating from different melanocytes that already harbor acquired driver mutations but are restrained from forming clinically manifest lesions by the immune system. An alternative mechanism is that lesions originate from a single melanocyte, whose daughter cells enter the circulation to seed multiple cutaneous sites, where they proliferate to spawn descendant nevi. Here we report a patient with widely disseminated Spitz nevi in which three distinct nevi were biopsied and all identified to harbor a *TPM3*-*ROS1* fusion, a known driver oncogene of Spitz nevi^2^. All three fusion genes had identical intronic breakpoints, showing that they originated from one single ancestral cell. To our knowledge, this is the first documented case of disseminated Spitz nevi demonstrating a clonal origin of their somatic mutations, providing strong support for the hypothesis that partially transformed melanocytes can circulate and seed spatially separated lesions as a cause of eruptive Spitz nevi. The term ‘eruptive Spitz nevus’ appears more apt for this setting.

## Results

**Figure 1:**
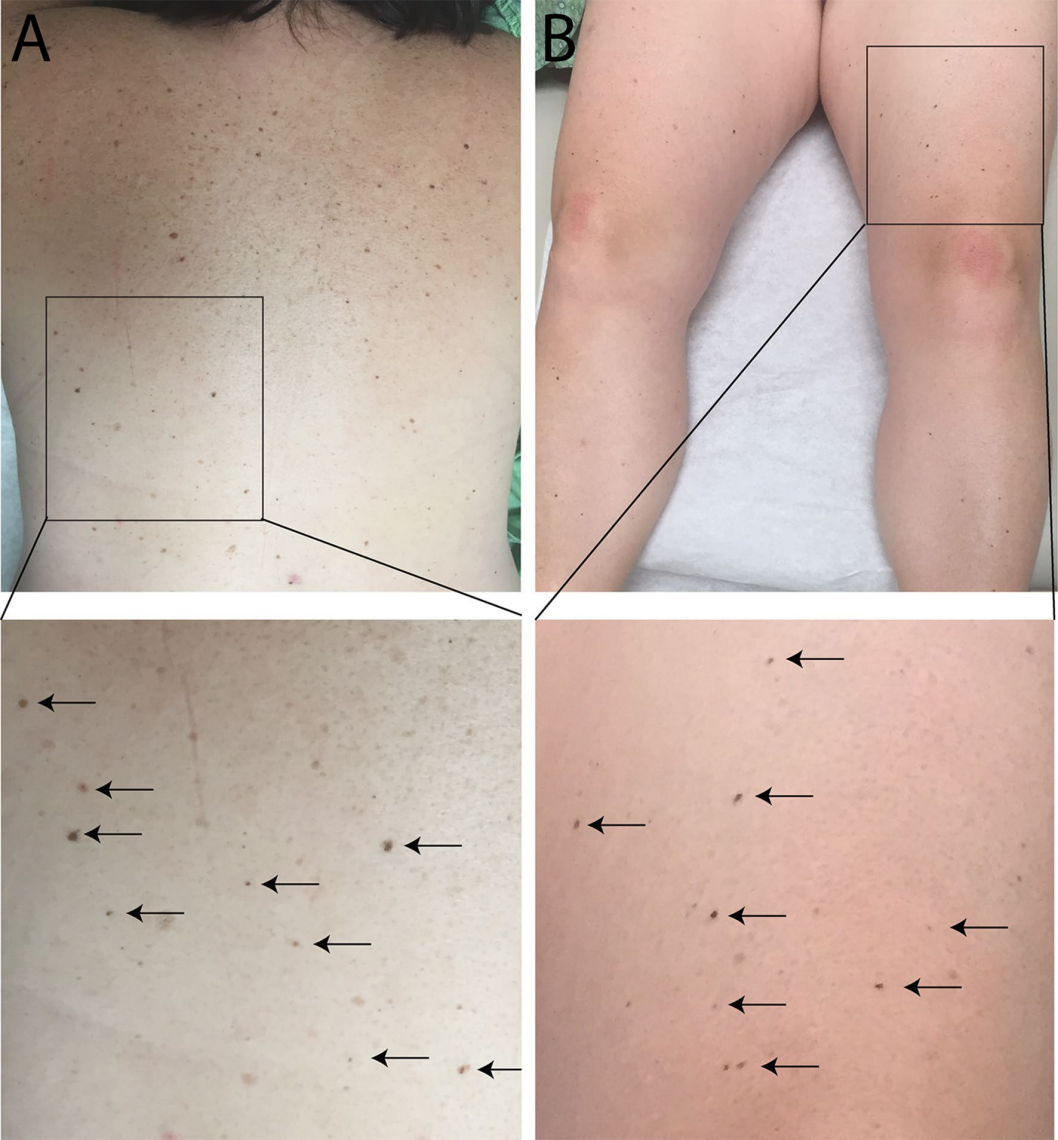
Clinical presentation of eruptive Spitz nevi. (**A**) TOP—Patient’s back with numerous small, dark brown papules. BOTTOM—Close-up view of the lower back shows discrete small round dark papules (arrows). (**B**) TOP—Similar dark brown papules are found on the patient’s legs. BOTTOM—Close-up view of the lesions on the left thigh.

A 49-year-old woman without a personal or family history of melanoma, presented for a full skin examination due to her history of basal cell carcinoma. A 3 mm in diameter pigmented papule on the left buttock was biopsied and diagnosed as a Spitz nevus. Histologically, the lesion was symmetric, well-circumscribed, and composed of spindled and epithelioid melanocytes present within the epidermis and dermis. There was epidermal hyperplasia, occasional scatter of melanocytes into the upper levels of the epidermis, and Kamino bodies. Melanin was visible within the cytoplasm of some melanocytes. The melanocytes had slightly enlarged nuclei, larger than keratinocyte nuclei with occasional nuclear pseudoinclusions. Occasional mitotic figures were present in the junctional component but absent in the dermal component. A diagnosis of Spitz nevus was rendered. Over the next 4 years, the patient developed more than 100 morphologically similar papules, each hyperpigmented and less than 5 mm in diameter. The new lesions first arose near the excision site of the initial lesion on the left buttock, but subsequently also emerged on the lower extremities and eventually on the trunk and upper extremities (Fig. 1).

**Figure 2:**
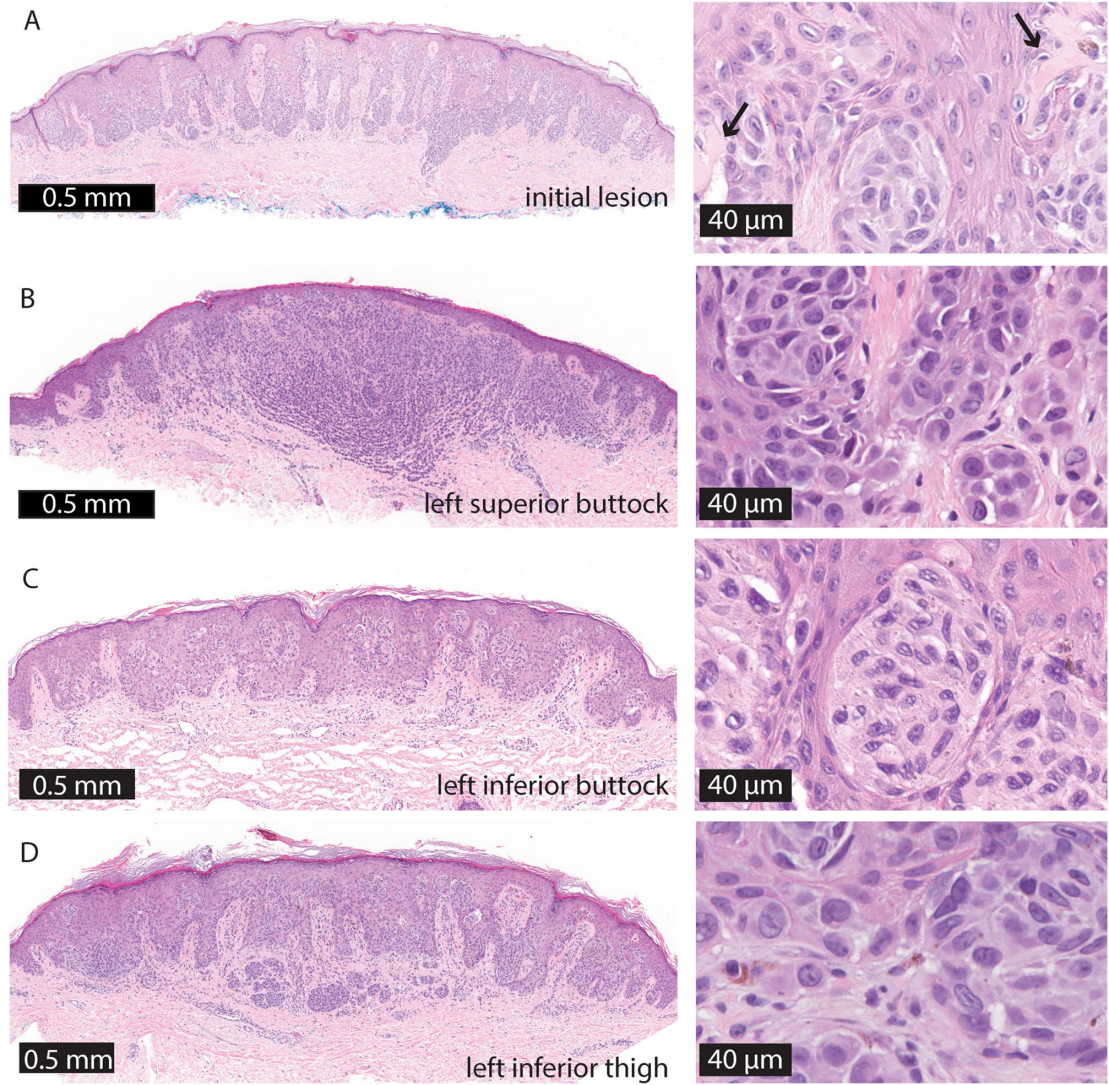
Representative histopathology of the eruptive Spitz nevi. (**A**) Initial lesion on left buttock. (**B**) Left superior buttock. (**C**) Left inferior buttock. (**D**) Left inferior thigh scanning magnification shows fairly well-circumscribed compound melanocytic proliferations with focal epidermal clefting and epidermal hyperplasia. Arrows indicate Kamino bodies. Higher magnification reveals variably sized nests of epithelioid melanocytes with abundant eosinophilic cytoplasm and occasional prominent nucleoli or intranuclear inclusions.

Fourteen of these new papules were biopsied and all showed Spitz nevi with similar histopathologic findings as in the original specimen (Fig. 2). Mitoses were rare in all specimens, with at most one dermal mitosis identified per high power field, indicating a low proliferation rate. Four years after the initial onset, the patient continues to develop additional pigmented papules at a similar rate and remains otherwise well. She was not on medications and did not have a history of immunosuppression.

**Figure 3:**
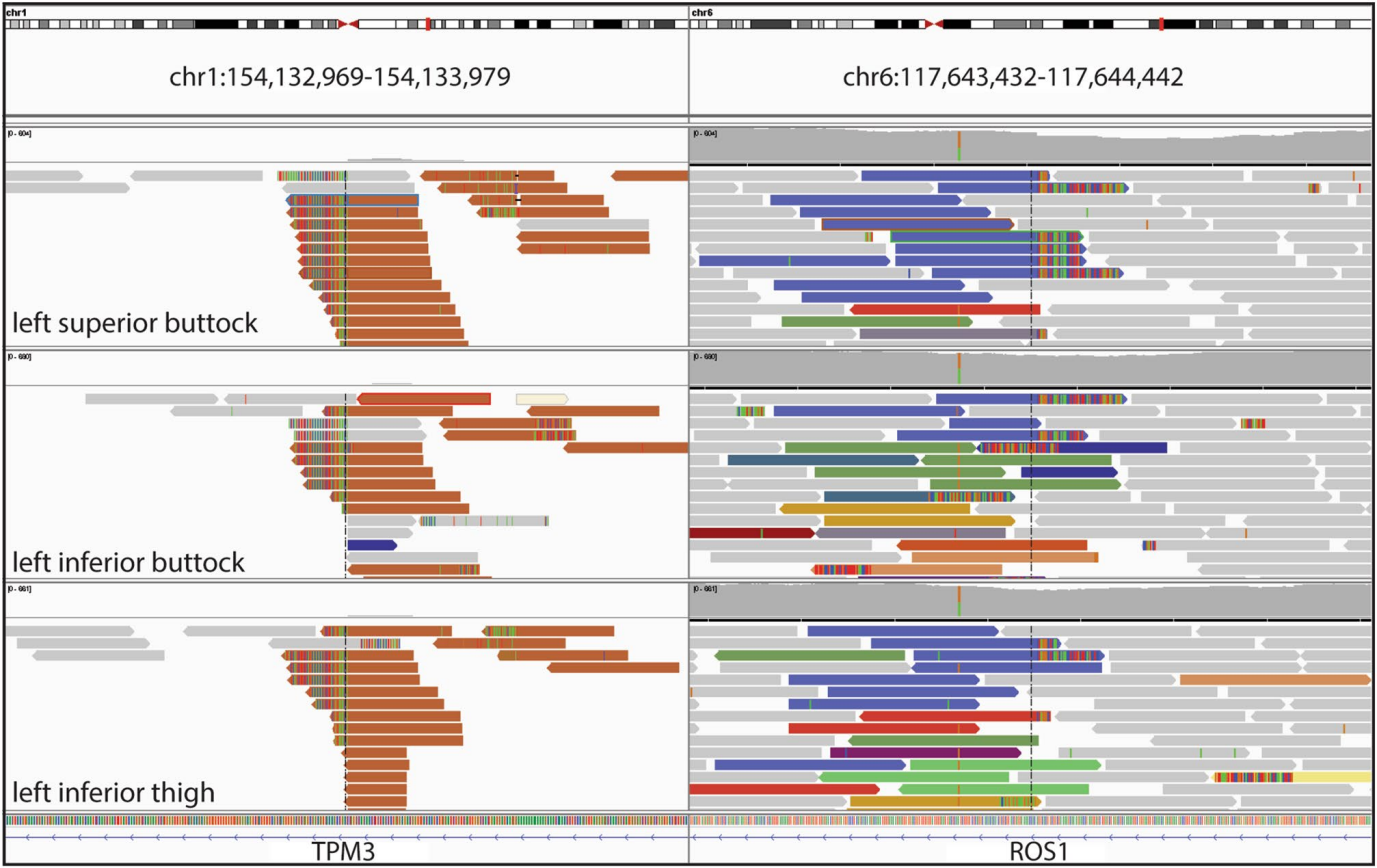
Identical *TPM3-ROS1* fusion in three Spitz nevi. Targeted sequencing of three separate lesions shows an identical fusion joining intronic sequences of *TPM3* and *ROS1*.

To determine the genetic relationship between individual Spitz nevi in this patient, we performed deep targeted sequencing of all exons and select introns of 480 cancer related genes for three of the Spitz nevi (left buttock superior, left buttock inferior, left thigh). All three Spitz nevi demonstrated a *TPM3*-*ROS1* rearrangement with identical intronic breakpoints (Fig. 3). Additional pathogenic mutations were not identified. We analyzed the genomic copy number of the tumors using the sequencing data as previously described^13^. The Spitz nevus from the left inferior buttock demonstrated copy loss of chromosome 12q, but no other copy number alterations. Copy number alterations were not identified in the other two Spitz nevi analyzed.

## Discussion

The kinase fusions that initiate Spitz nevi are thought to result from faulty repair of double-stranded DNA breaks within the introns of two separate genes, one encoding the kinase and the other its fusion partner, that become joined into one or more chimeric genes. The fusion partners can reside near each other on the same chromosome or on separate chromosomes. Because the breaks occur in the long stretches of non-coding DNA comprising introns of each of the fusion partners, a myriad of distinct rearrangements between the two genes are conceivable that can form a fusion gene that produces the exact same functional messenger RNA molecule after transcription and splicing. Thus, the probability of finding the exact same fusion junction in three separately initiated tumors of the patient is infinitesimally low, and the findings indicate that the tumors originated from a single cell that acquired the *TPM3*-*ROS1* fusion.

Several modes of dissemination could account for the distribution of lesions in our patient. Given that the first nevus appeared on the left buttock with subsequent nevi following nearby (i.e. forming agminated Spitz nevi), the initial transforming event may have occurred in a melanocyte or melanocyte precursor cell in this area. Two previously reported cases of eruptive Spitz nevi were also preceded by agminated Spitz nevi before wider dissemination^10,14^. In our case, as in other previously reported cases^9,15,16^, nevi first appeared on the lower extremities and buttocks before involving the trunk and upper extremities. Another consideration is that perhaps local dissemination first occurred due to migration along neurovascular bundles or via lymphatic vessels^17^. Peri- or intraneural invasion occurs in Spitz nevi and the ability of Spitz nevi to disseminate through the lymphatic vessels is supported by the finding of lymphatic invasion in more than 10%^18^. The ability of benign melanocytic tumors to travel through the lymphatic vasculature would account for the high incidence of sentinel lymph node metastasis of atypical Spitz tumors that is in striking contrast with an exceedingly low incidence of metastatic dissemination to distant sites in these patients^19^.

The fact that later lesions arose all over the integument in a symmetric distribution suggests that at one point the dissemination became hematogenous. In our case, as in other previously reported cases^9,15,16^, nevi first appeared on the lower extremities and buttocks before involving the trunk and upper extremities. Factors that would account for the initial appearance of disseminated nevi over the lower body are unknown but could be related to increased extravasation of tumor cells in dependent areas.

It is notable that the lesions were clinically monomorphous and involved the superficial skin. While it is impossible to know whether similar small neoplasms also are found in tissues beyond the skin, the stereotypical pattern of skin colonization suggests that trophic factors provided by the superficial skin promote the homing and proliferation of the transformed melanocytes necessary to form clinically visible lesions. Transformed cells that reach the skin by lymphovascular or hematogenous dissemination would need to actively migrate upwards through the skin and traverse the basement membrane zone to settle in the epidermis, thereby recapitulating the innately programmed path of melanocyte precursors during embryonal development. While the turnover of normal melanocytes in adult epidermis and the dynamics and location of relevant stem cell populations is not well understood, a stem cell population has been identified in the dermis that can give rise to epidermal melanocytes^20,21^. It is conceivable that eruptive Spitz nevi originate from such a stem cell by specific oncogenic alterations such a kinase fusion, giving rise to a partially transformed cell population with an increased proliferation rate that can maintain the typical homing pattern of its cell of origin, even after being swept away through the vascular system.

The molecular findings thus provide surprising insight into the pathogenesis of eruptive Spitz nevus. They represent a striking example of benign metastasis, in which a benign neoplasm disseminates widely throughout the body. This observation adds to the clinical observations that challenge the prevailing dogma that metastatic dissemination is a property that is acquired late in the evolution of primary neoplasms, constituting a defining property of malignancy. Instead, the findings indicate that neoplasms of certain lineages such as melanocytes can disseminate widely even at early stages, even if they are not malignant. The phenomenon of a benign tumor giving rise to a metastatic deposit has been termed benign metastasis, and it is not restricted to melanocytic neoplasia—other examples include benign uterine leiomyomas^22^, pleomorphic adenomas^23^, fibrous histiocytomas^24^, adenomyoepithelioma^25^, and meningioma^26^. The concept of benign metastasis is important to clinical care. The presence of tumor cells away from the primary site of a neoplasm is often interpreted as sufficient evidence that the neoplasm is malignant, even if the primary tumor lacks unequivocal features of malignancy. This can lead to unnecessary treatment, stigmatization, and morbidity.

## Methods

The study was approved by the human research ethics committee of UCSF (11-07922) and was conducted according to the Declaration of Helsinki and a waiver of informed consent was granted as no study procedures were performed. Microdissection of tumor tissue was performed from archival formalin fixed paraffin embedded (FFPE) tissue and DNA was isolated using standard laboratory procedures. DNA sequencing libraries were prepared with KAPA Hyper Prep Kit (KAPA Biosciences, Wilmington, MA p/n KK8504) according to the manufacturer’s instructions. A custom-designed bait library was used to target the coding regions of 480 cancer related genes (Nimblegen SeqCap EZ Choice, p/n 06588786001). Select introns including those of ALK, BRAF, MET, NTRK1, NTRK3, RET, and ROS1 were also targeted. Sequencing was performed as paired-end 100 bp reads on a HiSeq2500 (Illumina, San Diego, CA). Sequence analysis was performed as previously described^5^. Written informed consent for publication of photographs was obtained.
